# Super-resolution imaging of resonance modes in semiconductor nanowires by detecting photothermal nonlinear scattering

**DOI:** 10.1515/nanoph-2025-0383

**Published:** 2025-11-27

**Authors:** Yu-An Chen, Te-Hsin Yen, Chun-Yu Yang, Jhih-Jia Chen, Chih‐Wei Chang, Kentaro Nishida, Shi-Wei Chu

**Affiliations:** Department of Physics, 33561 National Taiwan University, 1, Sec 4, Roosevelt Rd., Taipei 10617, Taiwan; Center for Condensed Matter Sciences, 33561 National Taiwan University, 1, Sec 4, Roosevelt Rd., Taipei 10617, Taiwan; Center for Advanced Computing and Imaging in Biomedicine, 33561 National Taiwan University, 1, Sec 4, Roosevelt Rd., Taipei 10617, Taiwan; Brain Research Center, National Tsing Hua University, 101, Sec 2, Guangfu Rd., Hsinchu 30013, Taiwan

**Keywords:** super-resolution, photothermal effect, nonlinear, Mie resonance, dielectric nanophotonics

## Abstract

We demonstrated a far-field super-resolution optical imaging for mapping the resonance mode within semiconductor nanowires, where periodic distributions are found with good agreement between simulation and experiment. The pronounced absorption at the antinodes leads to localized photothermal heating, as well as consequent scattering nonlinearity via the thermo-optic effect. To break the diffraction limit, we combine the scattering nonlinearity with tightly focused laser scanning. Based on the principle of saturated excitation (SAX) microscopy, the nonlinear scattering signals are extracted to significantly improve the spatial resolution (1.7 fold), enabling visualization of the resonant modes that are not visible with conventional far-field optical imaging. Our results pave the way for optical inspection of semiconductor photonic integrated circuits with subdiffraction-limit spatial resolution.

## 1 Introduction

Semiconductor-based photonic integrated circuits have become foundational in advancing modern nanophotonic technologies, offering scalable and high-density platforms for manipulating light at the micro- and nanoscale [1], [2], [3]. These systems are constructed by a variety of high-index dielectric materials, which support sustaining optical resonant modes and enable strong light–matter interactions [4], [5], [6]. Among the various semiconductor platforms, silicon nitride (Si_3_N_4_) has emerged as a particularly promising material for the optical waveguide due to its wide transparency wavelength window [4], [6], low propagation losses [6], [7], and compatibility with standard CMOS fabrication processes, facilitating a broad range of applications, including optical communications [8], [9], nonlinear optics [10], quantum photonics [11], [12], and biosensing [13], [14].

To fully utilize the advantages of Si_3_N_4_-based photonic circuits, it is crucial to understand the resonance modes within the waveguide; specifically, characterizing the spatial distribution of optical fields and identifying the nature of supported modes is key to optimizing device performance. These insights inform the rational design of nanophotonic components and support the development of efficient and scalable photonic technologies. Currently, near-field scanning optical microscopy (NSOM) [15], [16], [17] and electron energy loss spectroscopy (EELS) imaging [18], [19] are the main tools for two-dimensional characterization of the electromagnetic resonant modes in semiconductor nanoresonators. Although these techniques have played important roles in the fundamental research to reveal the nanometric light–matter interaction, the drawbacks of the small field of view and the requirement of an expensive and complex measurement system make them less favorable for industrial in-line applications. On the other hand, far-field optical microscopy [20] offers noncontact, large-scale observation and better accessibility compared to NSOM and EELS systems. The well-known far-field resolution limit has been overcome via Mie-resonant photothermal nonlinearities recently, and successfully applied to inspect semiconductor nanowires in practical integrated circuits [21], [22]. However, its availability to characterize the fine distribution of the resonant modes in dielectric nanostructures has not been explored yet.

In this study, we developed a laser scanning-based far-field super-resolution microscopy to observe the resonance mode distribution induced within semiconductor nanowires. We experimentally confirmed the photothermal nonlinear scattering response localized at the antinode location of the periodic resonance modes within a single Si_3_N_4_ nanowire by using laser scanning microscopy (LSM). We extracted the nonlinear components of the scattering signal by applying saturated excitation (SAX) microscopy [23]. The spatial resolution improved 1.7 times by reconstructing the scattering image with the 3rd-order nonlinear scattering signal, and confirms our technique is able to resolve the resonance mode distribution in the semiconductor optical nanostructure beyond the resolution limit of a conventional far-field optical microscope.

## 2 Results

**Figure 1:**
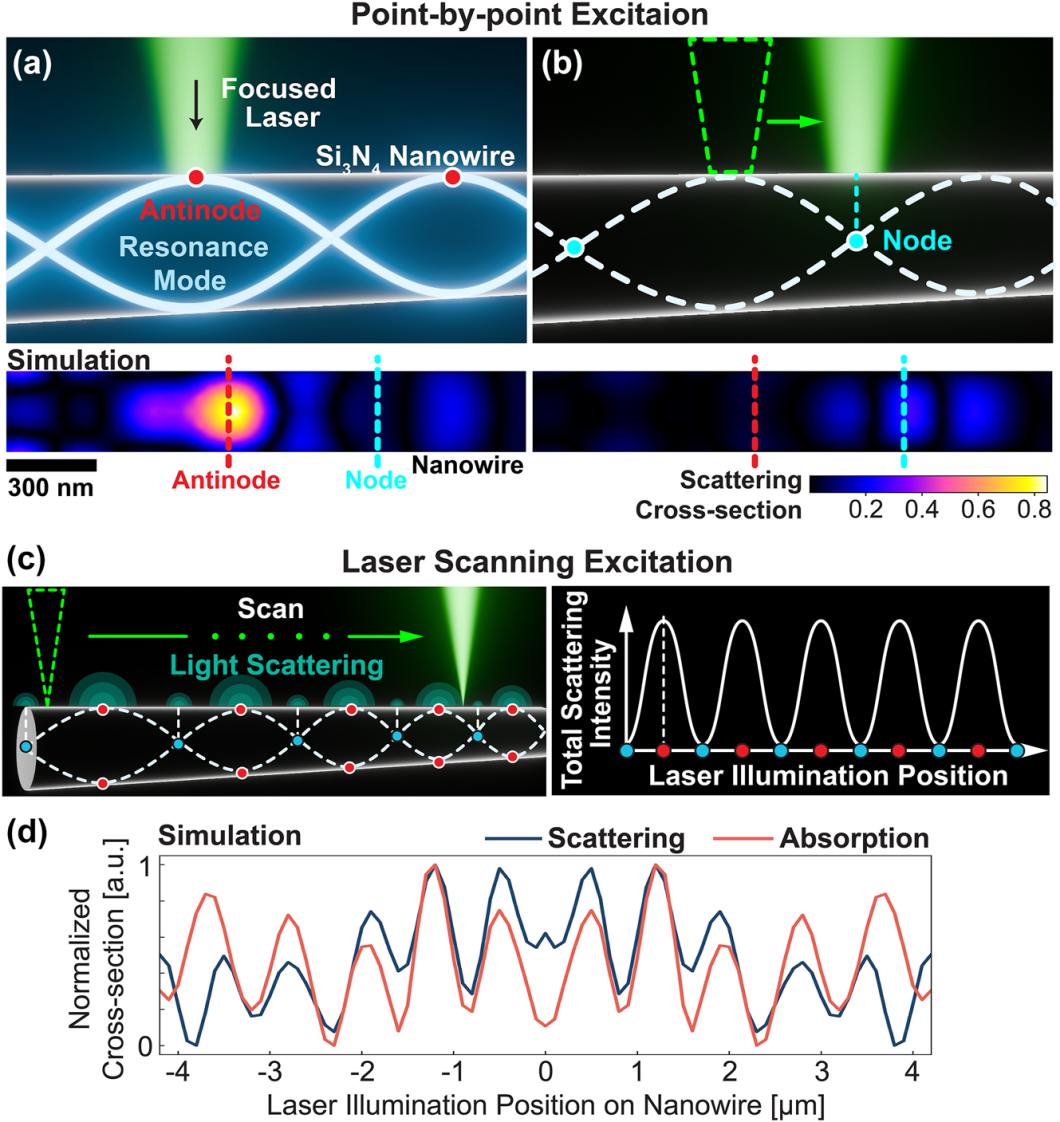
Laser scanning measurement of scattering signal from Si_3_N_4_ nanowire. (a, b) Schematics of a single Si_3_N_4_ nanowire illuminated with focused laser illumination, at the antinode (a) and node (b) locations of resonance modes, and corresponding numerical calculations of two-dimensional scattering cross-sectional distribution within the Si_3_N_4_ nanowire. The blue and red dotted lines in the simulation images indicate node and antinode locations, respectively. See Figure S1 of the Supplementary Materials for detailed calculation parameters. (c) Principle of laser scanning scattering measurement of Si_3_N_4_ nanowire. (d) Calculated dependencies of absorption and scattering cross section on laser illumination position of the Si_3_N_4_ nanowire. The blue and red solid lines indicate the scattering and absorption cross sections, respectively.

Figure 1a and b represents schematics to describe the optical response of a single Si_3_N_4_ nanowire under the point-by-point illumination of a focused laser beam. When a focused laser spot illuminates a dielectric nanowire whose diameter is comparable with the optical wavelength, the Mie resonant modes are excited at the illumination points [24], [25]. The localized Mie resonant mode couples with the intrinsic guided resonant mode of the nanowire by exploiting the nanowire as a Fabry–Pérot resonator, and a standing-wave pattern is induced, in turn modulating the light scattering/absorption properties [24], [26], [27]. Figure 1a presents that strong coupling and light scattering occur when the illumination spot is located at the antinode position, i.e., high local density of states, in the guided mode via the Purcell enhancement [26]. On the other hand, as shown in Figure 1b, when the laser spot moves to the node location where the local density of states is low, the coupling becomes weak with the low scattering cross section. The detailed simulation parameters and field distributions within the whole nanowire are described in Figure S1 of the Supplementary Materials.

Figure 1c shows the scheme of point-by-point scattering measurement across one nanowire via LSM, i.e., the situations of Figure 1a and b are alternately repeated during the beam movement. The finite-element method (FEM) simulation on the electric field distribution during laser scanning is given in Supplementary Video 1, manifesting that the total scattering intensity exhibits a periodic increase and decrease in response to the laser illumination position. That is, LSM should be able to observe the resonant guiding modes, as shown in the right schematic of Figure 1c, and confirmed by the simulation in Figure 1d, where scattering displays periodic patterns under LSM. Furthermore, Figure 1d also demonstrates that similar periodicity exists for absorption cross section, which modifies the subsequent photothermal behavior, leading to nonlinear scattering.

**Figure 2:**
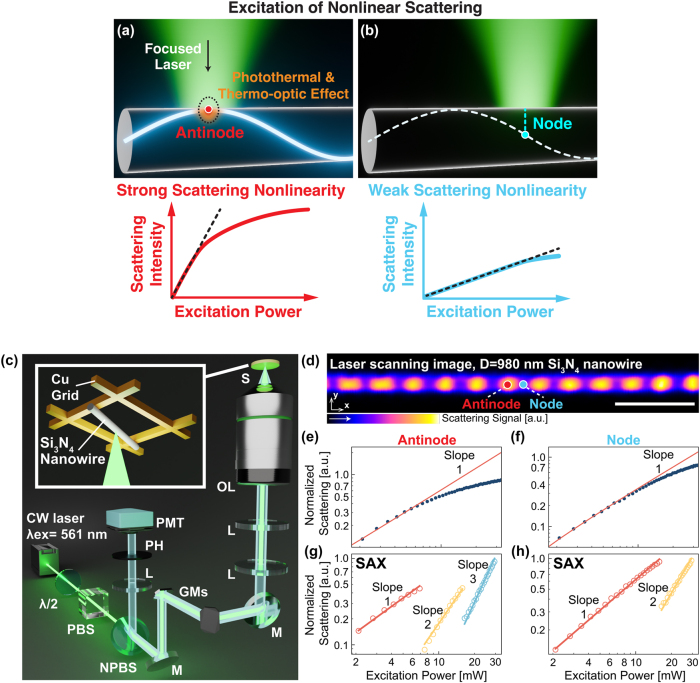
Measurements of the nonlinear scattering response from Si_3_N_4_ nanowire. (a, b) Si_3_N_4_ nanowire illuminated with the focused laser light at the antinode (a) and node (b) points, leading to the expectation of strong and weak scattering nonlinear responses, respectively. (c) Optical setup for laser scanning scattering microscopy and sample setup (CW: continuous-wave, *λ*/2: half-wave plate, PBS: polarizing beam splitter, NPBS: non-polarizing beam splitter, M: mirror, GMs: two-axis galvanometer mirrors, L: lens, OL: objective lens, S: sample, PH: pinhole, PMT: photomultiplier tube. (d) Laser scanning image of Si_3_N_4_ nanowire with a diameter of 980 nm. The excitation power is 5.5 mW at the sample. The scan speed is 0.79 frame/s. The scale bar is 5 μm. The red and blue dots indicate the antinode and the node points of signal distribution, respectively. (e, f) The relationships between the excitation power and experimentally measured scattering signals at the antinode (e) and the node (f), respectively. The solid red line indicates the linear slope. (g, h) The nonlinear scattering signal extracted by applying SAX microscopy, for the antinode (g) and the node (h) locations. Red, yellow, and blue plots are linear, 2nd-order, and 3rd-order scattering signals extracted from (e, f), respectively.

Figure 2a and b shows the idea of position-dependent nonlinear scattering in the Si_3_N_4_ nanowire by LSM. As shown in Figure 2a, when the focused laser aligns the antinode location, the photothermal effect is maximized. As a result, the relationship between the excitation power and scattering intensity exhibits a remarkable nonlinearity because the thermal variation of refractive index, i.e., thermo-optic effect, reduces the scattering cross section (see Supplementary Materials Figure S2 for the calculation results on the temperature-dependent scattering cross section of Si_3_N_4_ nanowire) [21], [28]. On the other hand, when the illumination position moves to the node, Figure 2b shows that the nonlinearity of scattering becomes relatively weak due to less absorption and photothermal effect.

We experimentally measured the nonlinear scattering signal of Si_3_N_4_ nanowires with LSM (Figure 2c). The light source was a continuous wave laser at 561 nm (Cobolt Jive, HÜBNER Photonics). The irradiation power of the light source was controlled by a pair of a half-waveplate and a polarizing beam splitter. The laser beam was focused onto the sample by an objective lens (UPlansApo 40×/0.95, Olympus), and raster scan was performed across the sample in two dimensions by controlling a pair of galvanometer mirrors with the pixel dwell time of 2.54 μs/pixel. The scattering signal from the sample was epi-collected by the same objective lens and detected by a photomultiplier tube (R9110, Hamamatsu Photonics) after a nonpolarizing beam splitter. The PMT gain was set to 1.38 × 10^−4^ A/lm with the supply voltage of 80 V. The background reflection and scattering signal was filtered by the combination of a lens and a pinhole equipped in the detection path. The size of the pinhole was set to 2.4 Airy Unit to improve the collection efficiency. The sample was prepared by attaching commercially available Si_3_N_4_ nanowires (silicon nitride fiber #806560, Sigma-Aldrich) onto a copper grid with a grid size of 50 µm in the atmospheric environment, as illustrated in the inset of Figure 2c. The diameters of the Si_3_N_4_ nanowires used in our experiments were confirmed by a scanning electron microscope, as shown in Supplementary Materials Figure S3. We verified the spatial resolution of our optical microscope system in Figure S4, by measuring the signal profile of the nanowire scattering image along the transverse direction, and comparing with the simulated signal profile.

Figure 2d is the laser scanning scattering image of one Si_3_N_4_ nanowire, whose diameter is 980 nm. As we expected, the distribution of the scattering signal exhibits periodic patterns along the direction of the wire, where the maximal and minimal signals correspond to the antinodes and nodes, respectively. In Figure 2e, i.e., the antinode point, the scattering intensity increases with a linear trend at the low excitation power regime. However, when the excitation power exceeds ∼8 mW, the scattering intensity starts to show saturation. On the other hand, in the case of the node location in Figure 2f, the nonlinearity of scattering occurs at the excitation power exceeding ∼12 mW, manifesting a higher threshold. In addition, the deviation of scattering intensity from the linear trend in Figure 2f is not as large as that of Figure 2e. These results confirm that the higher absorption at the antinode locations (Figure 1d) more efficiently induces the scattering nonlinearity than that at the node location, supporting the mechanism of photothermal and thermo-optic Mie resonance shift [21], [28] (see Supplementary Materials Figure S2 for the calculation results on the temperature-dependent scattering cross section of Si_3_N_4_ nanowire).

It is well known that nonlinearity leads to resolution enhancement. Here, we extracted the nonlinear components in the scattering signal by applying the idea of differential SAX (dSAX) microscopy [29], as briefly described below. Although dSAX microscopy was originally developed for fluorescence imaging, the fundamental principle of dSAX is directly applicable to nonlinear scattering imaging by modifying the detection modality. Specifically, the fluorescence dSAX microscope system uses a dichroic filter before the photodetector to selectively collect fluorescence signals, whose wavelength is longer than the excitation. In contrast, our scattering dSAX microscopy detects scattering signals, the same wavelength as the excitation, by a 50/50 nonpolarizing beam splitter instead of wavelength filters (Figure 2c).

Without losing generality, the scattering nonlinear response is expressed as a polynomial expansion:

(1) $$S\left(I_{\text{ex}}\right)=a_{1}I_{\text{ex}}-a_{2}I_{\text{ex}}^{2}+a_{3}I_{\text{ex}}^{3}-\cdots$$

where *S* is the total scattering intensity, *a*_*n*_ (*a*_*n*_>0) is the coefficient of the *n*_th_ order scattering signal, and *I*_ex_ is the excitation intensity. The 2nd-order nonlinear signal *S*_2ndNL_ is extracted by calculating the difference between the linear scattering signal and the total scattering signal:

(2) $$\begin{array}{c} S_{2\text{ndNL}}\left(I_{\text{ex}}\right)=a_{1}I_{\text{ex}}-S\left(I_{\text{ex}}\right)\;=a_{2}I_{\text{ex}}^{2}-a_{3}I_{\text{ex}}^{3}+\cdots \end{array}$$

Note that, although other nonlinear components higher than the 2nd order are included in *S*_2ndNL_, these higher order components are typically negligibly low, and thus the 2nd-order nonlinear signal is dominant in *S*_2ndNL_. Following the same principle, the 3rd-order nonlinear component *S*_3rdNL_ is obtained by subtracting the 2nd-order nonlinear components from Eq. (2):

(3) $$\begin{array}{c} S_{3\text{rdNL}}\left(I_{\text{ex}}\right)=a_{2}I_{\text{ex}}^{2}-S_{2\text{ndNL}}\left(I_{\text{ex}}\right)\;=a_{3}I_{\text{ex}}^{3}-\cdots \end{array}$$

In Figure 2g, the nonlinear components at the antinode location are extracted from Figure 2e. The red circles present the 1st-order linear components, while the yellow and blue circles are 2nd-order and 3rd-order nonlinear scattering contributions, featuring square and cubic power dependencies, respectively (see Supplementary Materials Figure S5 for detailed calculation parameters of dSAX microscopy). The nonlinear power dependency is the basis of subsequent super-resolution inspection. The 2nd-order and 3rd-order nonlinear signals lead to the reduction of the point spread function (i.e., the improvement of spatial resolution) by a factor of √2 and √3, respectively, in laser scanning microscopy [23], [30]. On the other hand, at the node location, where the absorption and scattering nonlinearity is relatively weak, Figure 2h shows that the nonlinear signal component higher than the 2nd order is not detected.

**Figure 3:**
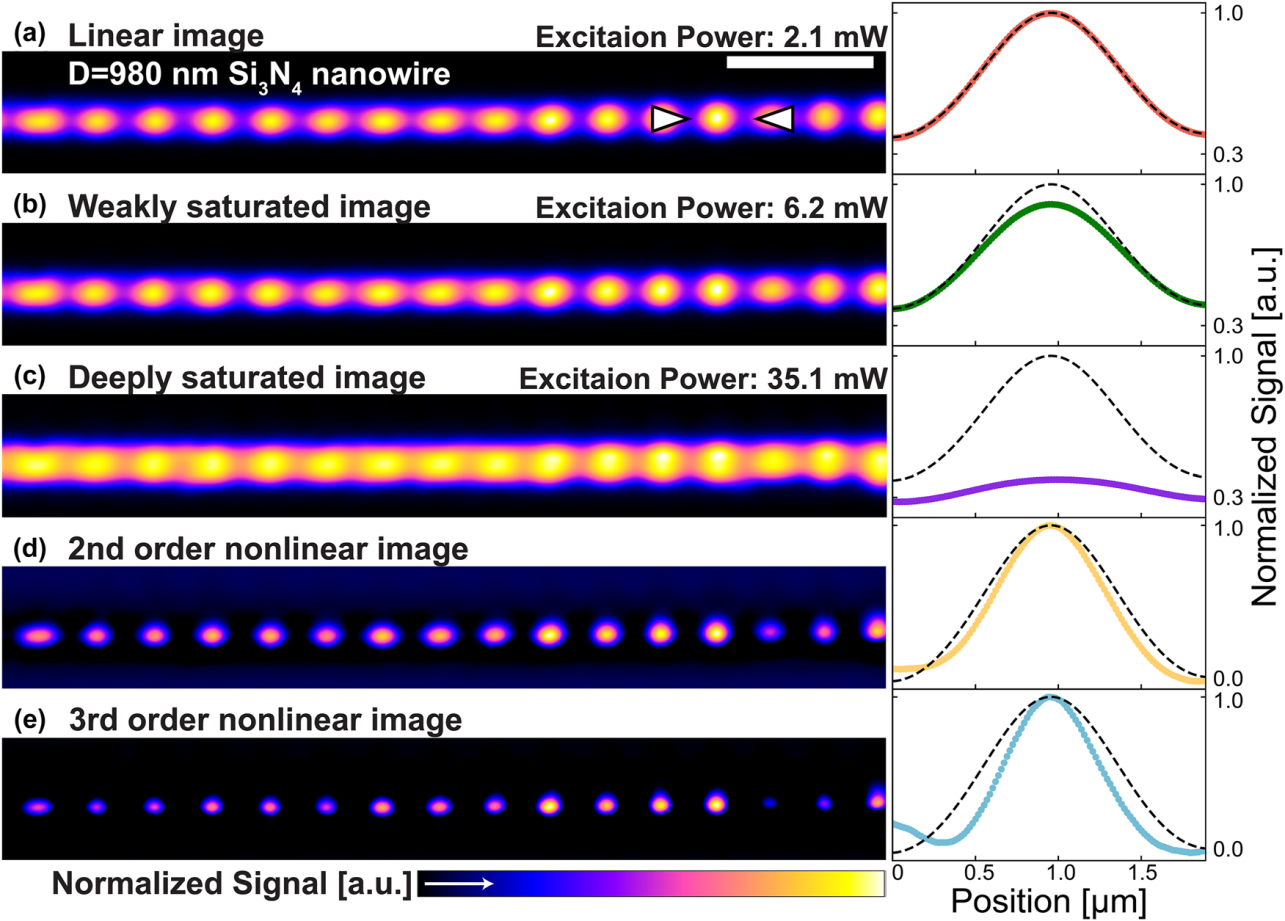
Super-resolution images of 980 nm diameter Si_3_N_4_ nanowire obtained by SAX microscopy. (a–c) Linear and saturated scattering images of Si_3_N_4_ nanowire with a diameter of 980 nm. The scale bar is 5 µm. (d, e) Scattering SAX images reconstructed by 2nd- (d) and 3rd-order (e) nonlinear signal. The corresponding signal profiles obtained at the position of white arrowheads in (a) are attached to the right side of each image, where the black dotted lines indicate the linear profiles. Excitation powers are 2.1 mW (a), 6.2 mW (b, d), and 35.1 mW (c, e) at the sample. The scan speed is 0.79 frame/s.

Figure 3a–c shows the laser scanning scattering images of the Si_3_N_4_ nanowire with a diameter of 980 nm, acquired at increasing excitation powers from 2.1 mW, 6.2 mW, to 35.1 mW, manifesting clear saturation. Their corresponding longitudinal signal profiles are presented on the right side of each image, together with the black dashed lines to represent linear signal profiles. In Figure 3a, when the excitation power is low, the scattering signals match the linear profile well, while in Figure 3b, slight saturation appears from the center of the image, where the absorption and photothermal effect are strongest. In Figure 3c, significant saturation under high excitation power is manifested by a large deviation from the linear profile.

Figure 3d and e shows the scattering images reconstructed by the 2nd- and 3rd-order nonlinear signals via SAX microscopy, demonstrating obvious resolution enhancement. Quantitatively, the full width at half maximum (FWHM) of the signal profiles in linear, 2nd-order, and 3rd-order nonlinear images gradually reduces as 928 ± 38, 677 ± 14, and 527 ± 8 nm. The spatial resolution was improved by factors of 1.37 and 1.76 in second- and third-order SAX imaging, respectively, which are in close agreement with the theoretical predictions of 1.41 and 1.73. The slight deviations are likely attributable to residual optical aberrations in the scanning system illustrated in Figure S4b or to structural imperfections of the Si_3_N_4_ nanowire depicted in Figure S3b. These results confirm that the selective detection of the nonlinear scattering signal by SAX microscopy allows us to precisely map the locations of the antinode inside the nanowire with high spatial resolution.

It should be noted that nonuniformities of the signal intensity in each antinode location of the resonant mode are observed in both linear and SAX images, probably because the diameter of the nanowire is nonuniform, thus resulting in the variation of the scattering efficiency and nonlinear response at each illumination position. Another remark is that during subtraction, high spatial frequency noise/artifact might arise, and a low-pass filter was applied to remove it. The detailed information of the low-pass filter is shown in Supplementary Figure S6.

**Figure 4:**
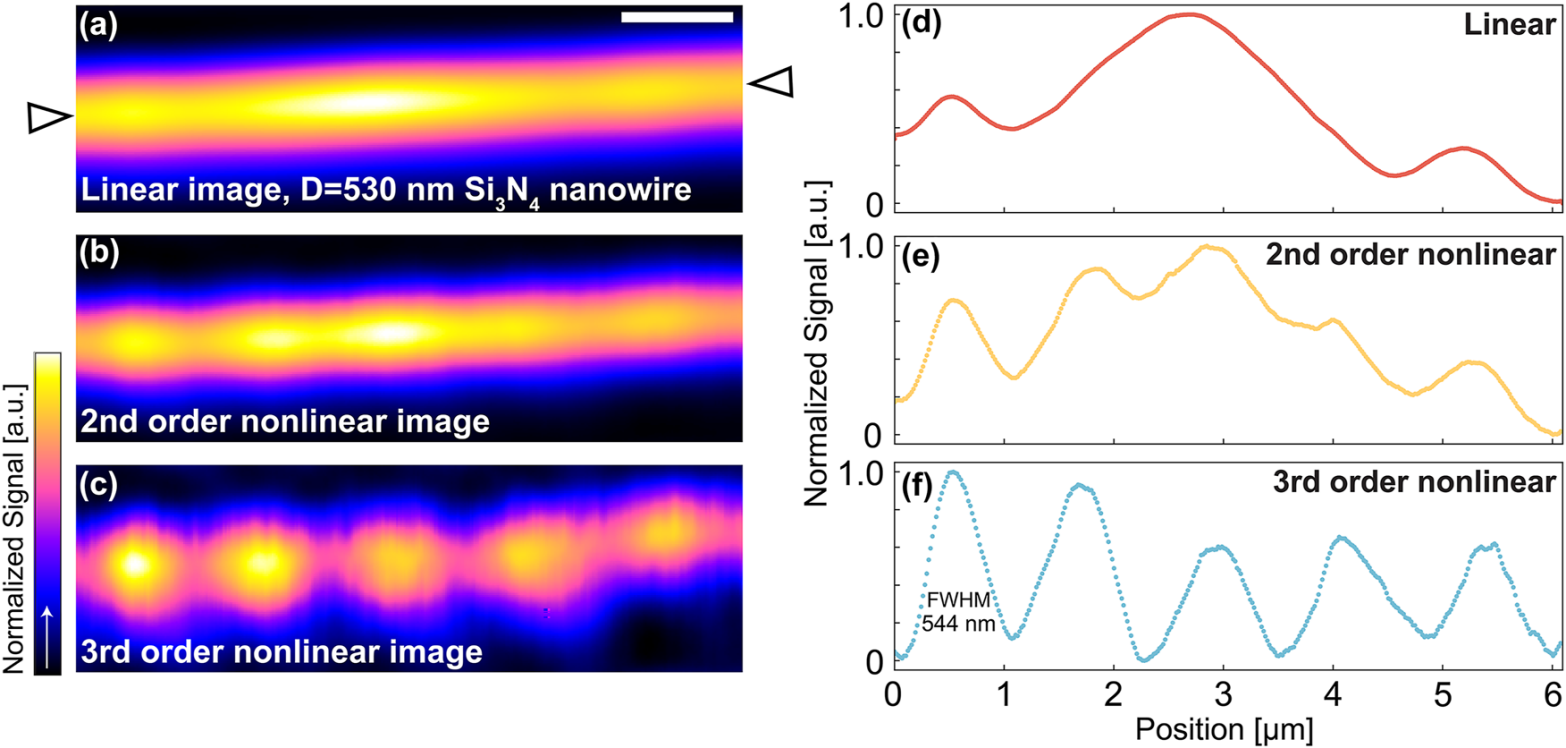
Super-resolution images of 530 nm diameter Si_3_N_4_ nanowire obtained by SAX microscopy. (a–c) Scattering images of Si_3_N_4_ nanowire with a diameter of 530 nm, reconstructed by linear (a), 2nd- (b), and 3rd-order nonlinear signal (c). Excitation powers are 2.8 mW (a), 13.1 mW (b), and 24.1 mW (c) at the sample. The scan speed is 10 frame/s. The scale bar is 1 µm. (d–f) Signal profiles of scattering images (a–c), obtained along the white arrowheads in (a).

To demonstrate the super-resolution capability, i.e., unveil the features that are not visible at low power, we observed another Si_3_N_4_ nanowire with a smaller diameter (530 nm), whose resonant modes exhibit a shorter period than the 980 nm diameter one (see Supplementary Materials Figure S7 for the simulation results of scattering distribution). Figure 4a is the linear image, where the density of optical modes is too high to be resolved. The periodic modes of the nanowire are only visible in the nonlinear super-resolved images of Figure 4b and c; in particular, the 3rd-order nonlinearity delivers the best resolution.

One additional remark is that the linear and nonlinear signal strengths might be different. For example, the maximal signal values of Figure 4d and e appear at the position of ∼3 µm, but in Figure 4f, the peak signal at the same position is no longer maximal. This discrepancy arises because the spatial distributions of scattering and absorption are not perfectly aligned, i.e., the regions of maximal scattering do not coincide with maximal photothermal saturation, as manifested in Figure 1d.

## 3 Conclusions

In summary, we developed a far-field super-resolution optical imaging technique to map the resonance modes within semiconductor nanowires. Our FEM simulation reveals that under laser scanning illumination, a unique periodic scattering distribution exists in Si_3_N_4_ nanowires, and through investigating the relationship between excitation power and scattering intensity of the nanowire under LSM, we experimentally measured the photothermal nonlinear scattering response that is maximized at the antinode locations. The super-resolution inspection was realized via SAX microscopy, which extracted the nonlinear components of the scattering signal to reconstruct the scanning image, and we quantitatively demonstrated 1.4- and 1.7-fold improvements of the spatial resolution with the 2nd- and 3rd-order nonlinear images, respectively. The technique allows us to examine the periodic resonance modes that are not visible in the linear modality, but only resolvable with the nonlinear signals.

This is the first demonstration of super-resolution imaging of resonance mode pattern in semiconductor nanostructure using a far-field optical method, to the best of our knowledge. Our technique enables noncontact inspection under ambient conditions, without the need for a vacuum environment or complex instrumentation unlike existing imaging modalities such as NSOM and EELS, and provides a more accessible alternative for studying resonance phenomena. In particular, the method is well-suited for investigating thermally responsive optical materials, photonic resonators, and *in situ* resonance behavior under ambient conditions. On the other hand, the spatial resolution currently achieved by our technique remains on the order of a hundred nanometers, which has not yet reached the levels of conventional imaging modalities. Typical resolutions are 20–100 nm for NSOM [15], [16], [17] and around 1 nm for EELS [18], [19]. Therefore, the next step of our research should be on the effort to further improve the spatial resolution of dSAX microscopy.

In principle, the spatial resolution of SAX microscopy is unlimited by detecting high-order nonlinear components of the scattering signal; for example, a 10-fold improvement of spatial resolution was demonstrated in SAX imaging of gold nanoparticles [31]. Nevertheless, in practice, the resolution is limited by signal-to-noise ratio (SNR), because the photon budget of the SAX signal rapidly degrades as the nonlinear order becomes higher [29], and the influence of shot noise is amplified. We evaluated SNRs in our technique by applying the Fourier transformation to the obtained dSAX images, as discussed in Figure S8 in detail. The SNR of the first-through third-order nonlinear images reduced quickly from 89.0, 11.2 to 9.5, respectively. While one straightforward method to enhance SNR is to increase the excitation power or the image acquisition time, the fundamental constraint is the thermal damage threshold. Under standard atmospheric conditions, Si_3_N_4_ nanowires exhibit exceptional thermal stability, decomposing above ∼1,873 K [32], which surpasses the melting or decomposition points of common semiconductors such as Si, Ge, GaAs, InP, and CdTe [33]. In our experiments, no evidence of thermal degradation in the nanowire structure was observed, even at our highest excitation power approaching ∼60 mW. This conclusion was supported by repeated and reversible measurements on the same nanowire, performed across a range of excitation powers up to 60 mW. To further validate the thermal stability of Si_3_N_4_ nanowires under high excitation, techniques such as Raman thermometry could be employed in future studies to directly measure the local temperature [34], [35]. More efforts to enhance the thermal stability of the sample would offer a possible path to access even higher-order nonlinear scattering response and thus higher spatial resolution; for example, enriching the ambient environment with inert nitrogen may elevate the decomposition point to 2,775 K [36]. In addition, the use of temporally modulated excitation beams, such as pulsed lasers, potentially mitigates heat accumulation by introducing relaxation intervals, which facilitates the detection of higher-order nonlinear signals. Temporal modulation also enables control over the heat diffusion length, confining photothermal signal generation to narrower regions and consequently improving the spatial resolution of SAX imaging. Therefore, integrating differential SAX microscopy with pulsed excitation may represent an effective strategy to further enhance the performance of our technique.

Emerging computational technologies such as quantum computing and photonic computing increasingly rely on photonic integrated circuits composed of semiconductor nanowaveguides [37], [38]. Advancing this field requires not only improved fabrication techniques but also the development of inspection methods capable of analyzing the electric field distribution and optical resonance modes within these waveguides, as these properties are closely tied to their propagation behavior and functionality. Our method provides access to these mode patterns with the advantages of label-free and far-field, offering direct evaluation of mode confinement, coupling efficiency, and scattering losses across intact devices. Such information is critical for identifying fabrication-induced asymmetries, verifying optical connectivity among components, and diagnosing resonance shifts that affect circuit performance. In this way, our technique offers a complementary inspection pathway to address both geometry and functional optical behavior, beyond purely structural measurements. This dual capability is particularly valuable in large-scale manufacturing of semiconductor photonic integrated circuits, where it is important to ensure not only dimensional accuracy but also uniform optical properties across dense circuits.

## Supplementary Material

Supplementary Material Details
