# Supplementary material for: Super-resolution imaging of resonance modes in semiconductor nanowires by detecting photothermal nonlinear scattering
Source: Nanophotonics. 2025 Nov 27;14(25):4565–73. doi: 10.1515/nanoph-2025-0383 (PMC12714065; doi:10.1515/nanoph-2025-0383)
Supplement: Supplementary file 1 — Supplementary Material Details [file j_nanoph-2025-0383_suppl_001.pdf]

Supplementary Materials:

Super-resolution imaging of resonance modes in semiconductor nanowires by detecting photothermal nonlinear scattering

Yu-An Chen[1], Te-Hsin Yen[1], Chun-Yu Yang[1], Jhih-Jia Chen[1], Chih-Wei Chang[2], Kentaro Nishida[1]* and Shi-Wei Chu[1,3,4]*

[1]Department of Physics, National Taiwan University, 1, Sec 4, Roosevelt Rd., Taipei 10617, Taiwan

[2]Center for Condensed Matter Sciences, National Taiwan University, 1, Sec 4, Roosevelt Rd., Taipei 10617, Taiwan

[3]Center for Advanced Computing and Imaging in Biomedicine, National Taiwan University, 1, Sec 4, Roosevelt Rd., Taipei 10617, Taiwan

[4]Brain Research Center, National Tsing Hua University, 101, Sec 2, Guangfu Road, Hsinchu 30013, Taiwan

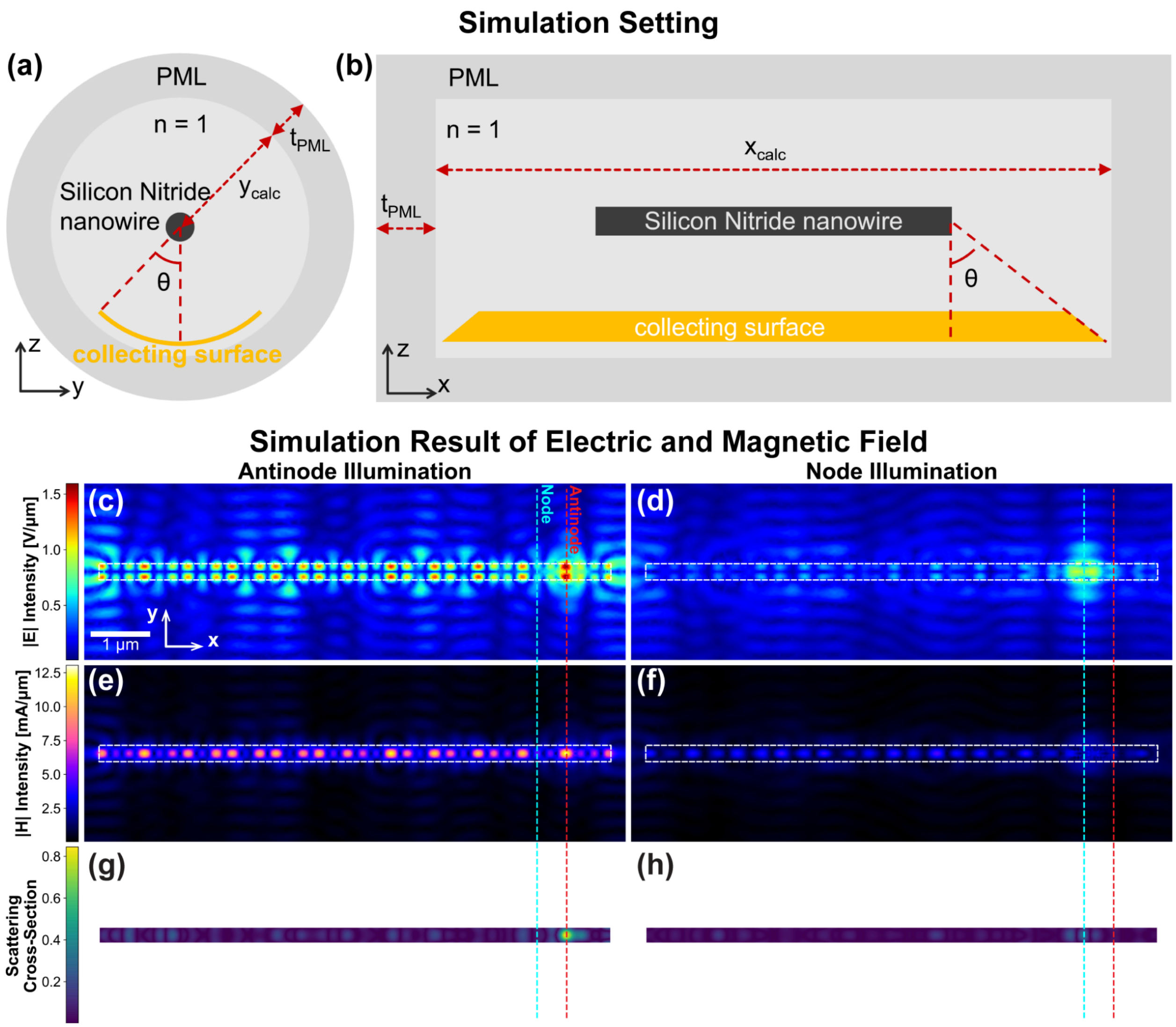

Figure S1: Simulation setup for the finite element method. t_{PML} is the thickness of the perfectly matched layer (PML). x_{calc} and y_{calc} are the length and the radius of the calculation domain. (a) Y-Z plane view of the simulation structure. (b) X-Z plane view of the simulation structure. All the simulation data is produced by the RF module of FEM-based software COMSOL Multiphysics (COMSOL Inc.). The silicon nitride nanowire with 276.2 nm diameter and 8.70 μm length is placed in the center of the calculation domain, which is a cylinder with a 1.10 μm radius and 10.7 μm length. In order to eliminate boundary reflection, the calculation domain was surrounded by a PML with a thickness of 561 nm, which corresponds to the excitation wavelength. The real and imaginary refractive indices of the silicon nitride were set to 2.0311 and 0. The refractive index of the surrounding medium and PML was set to be n = 1.00 to correspond with the air in the experiment. The incident light is set to be a linearly y-polarized focused Gaussian beam with a wavelength of 561 nm in free space, propagating in +z direction. The scattering cross-section in laser scanning is calculated by integrating the Poynting vector over a monitor surface divided by incident intensity. In order to mimic the experimental

condition, the monitor surface is set to be a columnar surface whose azimuthal angle θ corresponds to the NA of the objective lens, which is 0.95 in our case, and its length is longer than the wire at both ends, which is also determined by the NA of the objective lens. (c) X-Y plane view of the electric field intensity when the laser illumination position is at the antinode, which is marked by the red dot line. And the wire position is marked by the white dot box. (d) X-Y plane view of the electric field intensity when the laser illumination position is at the node, which is marked by the blue dot line. (e,f) X-Y plane view of the magnetic field intensity when the laser illumination position is at the antinode (e) and node (f). (g,h) X-Y plane view of the scattering cross-section distribution when the laser illumination position is at the antinode(g) and at the node (h). (c)-(h) use the same scale bar and axis labeled in (c).

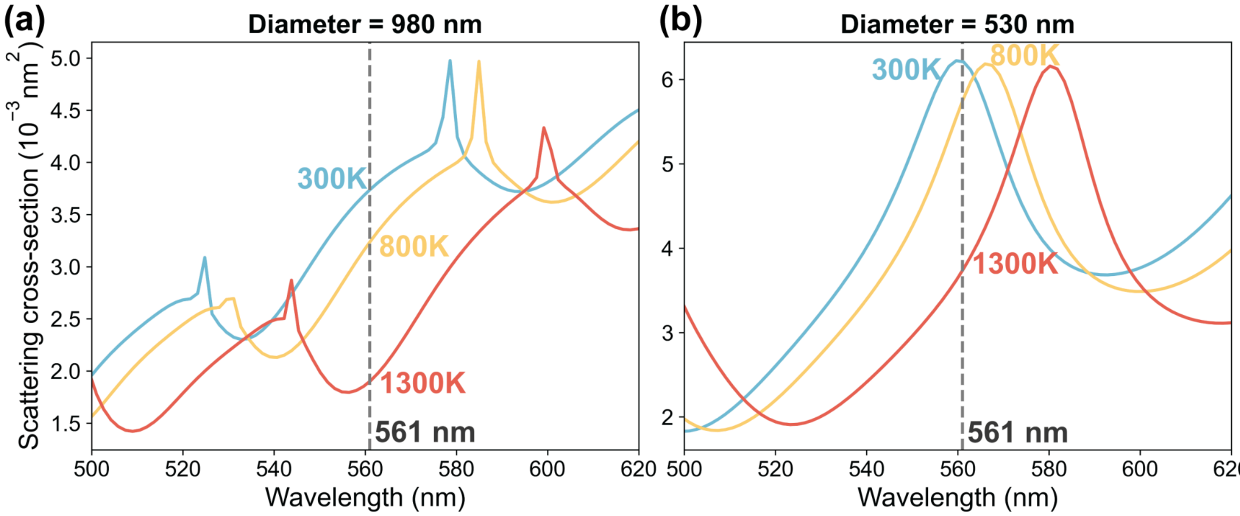

Figure S2. Calculated temperature-dependent scattering cross-section spectrum of Si_3N_4 nanowire obtained by the analytical solution, for (a) 980 nm diameter and (b) 530 nm diameter. These spectra are calculated by applying the analytical solution of Mie theory for an infinite length of cylindrical structure based on Chapter 8 of Ref (1). The temperature-dependent refractive index of Si_3N_4 is derived by the fitting model $n = aT^2 + bT + c$, where a is 6.5×10^{-8} K^{-2} (Ref (2)), b is -1.95×10^{-5} K^{-1} (Ref (2)), and c is 2.0311 (Ref (3)). The extinction coefficient is set to $k = 0$ because it is negligibly low in the range of 0.1 to 1 ppm at the wavelength range of 532 nm to 1550 nm (Ref (4)).

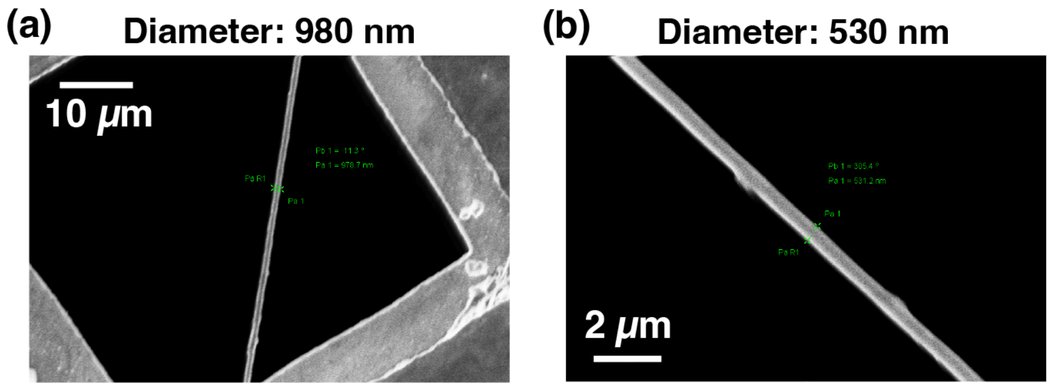

Figure S3: Confirmation of the diameters of Si_3N_4 nanowires used in the experiments by SEM. (a,b) SEM images of Si_3N_4 nanowires with the diameters of 980 nm (a) and 530 nm (b).

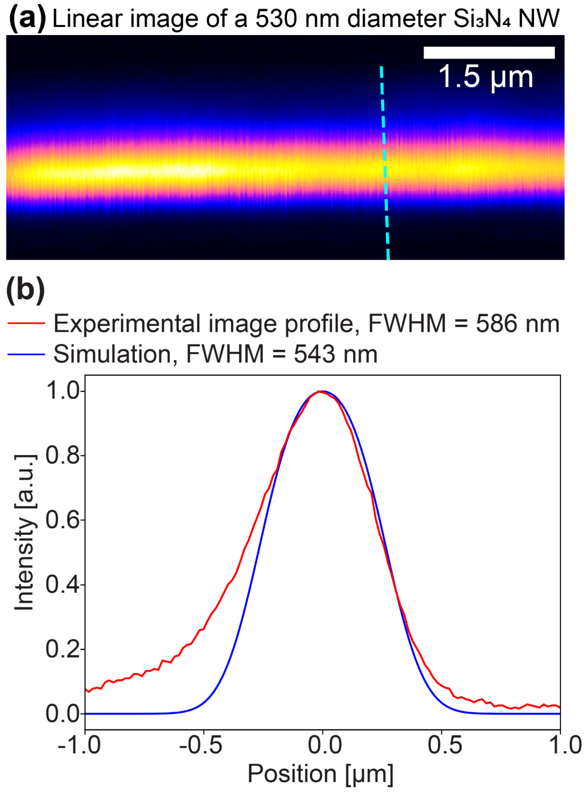

Figure S4: Comparison of spatial resolution with the simulation. (a) Linear scattering image of Si_3N_4 nanowire with a diameter of 530 nm. The excitation power is 2.79 mW. (b) Comparison of experimental image profile with simulation. The red solid line indicates the signal profile of an experimental scattering image measured at the dotted line in (a). The blue solid line indicates the simulated image profile, calculated by the convolution between step function with 530 nm width and Gaussian function with the FWHM of $0.51\lambda/NA$ = 301 nm, where the free space wavelength λ=561 nm and the numerical aperture NA=0.95.

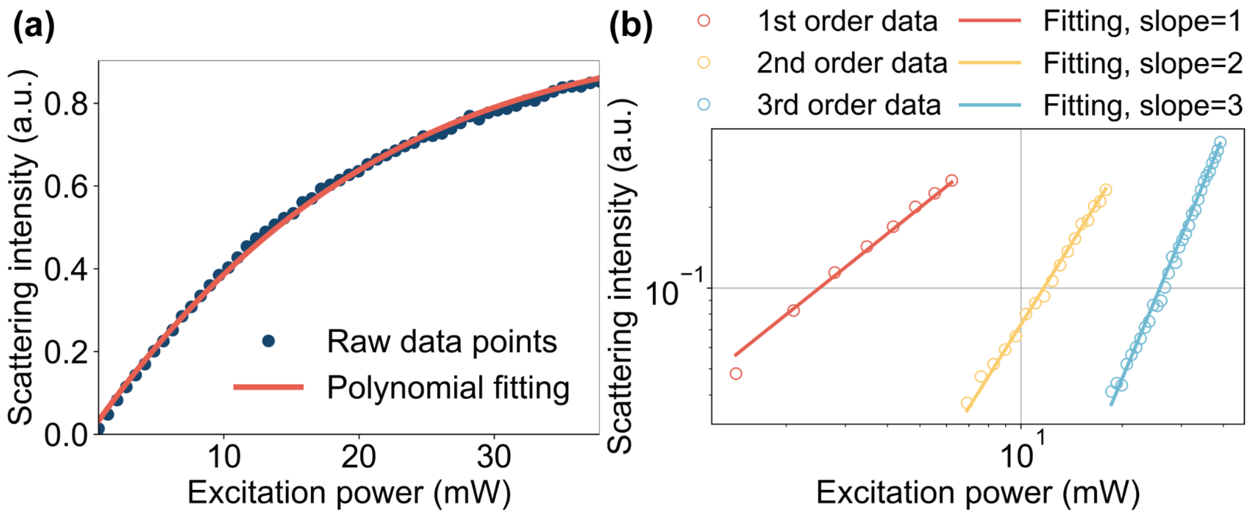

Figure S5. Extraction of nonlinear scattering signal from the antinode point of the silicon nitride nanowire with a diameter of 530 nm by SAX microscopy. (a) Polynomial fitting on the relationship between excitation power and scattering intensity. The blue dots and red curve respectively indicate the raw data and fitting curve. The fitting curve is 3rd order polynomial curve: $S(I) = a_0+a_1I+a_2I_2^2+a_3I_3^3$, and each constant values obtained by fitting a_0, a_1, a_2, a_3 are -1.57×10^{-3}, 4.67×10^{-2}, -8.54×10^{-4} and 5.86×10^{-6}, respectively. (b) The red, yellow, and blue plots indicate the linear, 2nd-order nonlinear, and 3rd-order nonlinear signals extracted by using Eqns. 1 - 3 in the main manuscript.

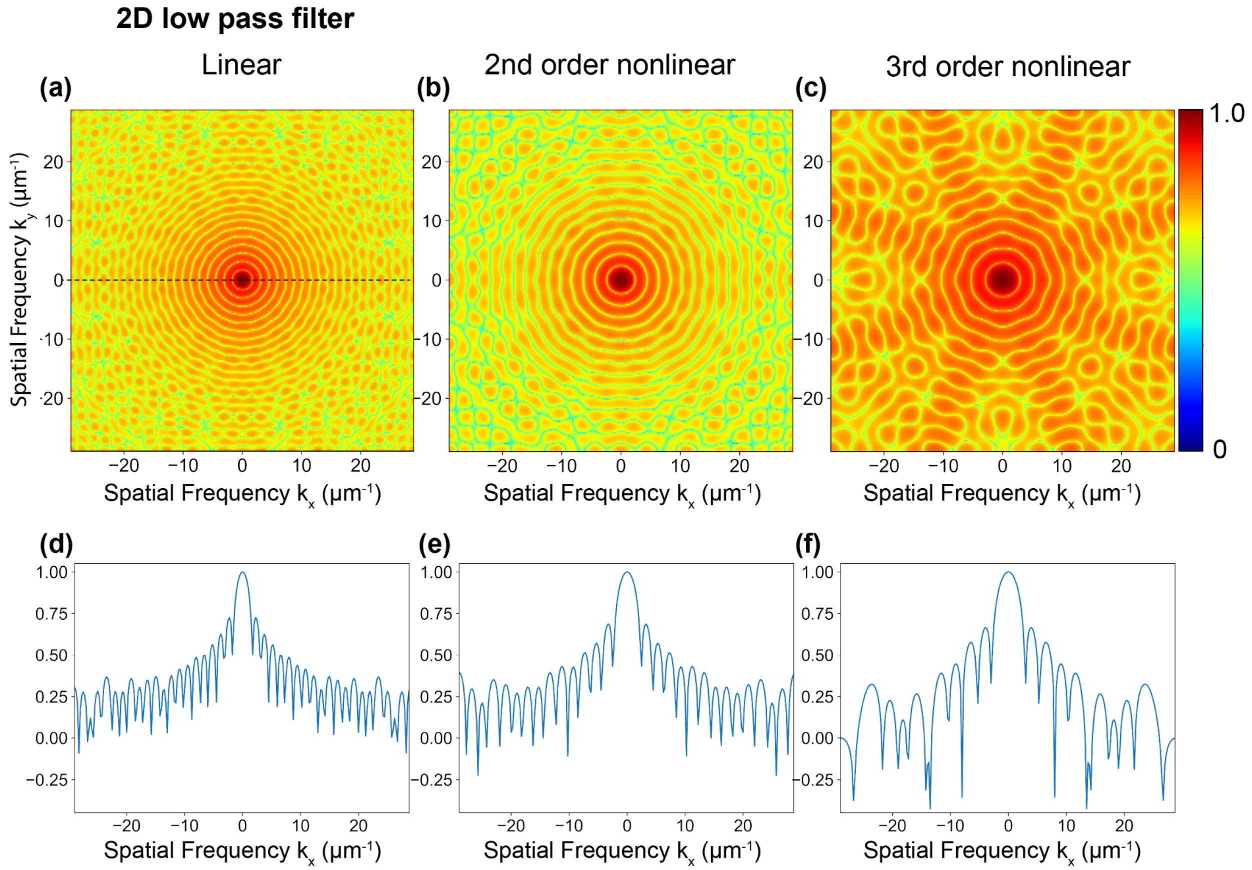

Figure S6. Low-pass filters used for denoising. (a-c) Two-dimensional functions of a low-pass filter to denoise linear (a), 2nd order nonlinear (b), and 3rd order nonlinear (c) dSAX images in spatial frequency domains of x (k_x) and y (k_y) coordinates in our research. These filter functions were calculated by applying the Fourier transform to 2D circularly symmetric aperture function whose diameters correspond to the effective point spread functions of our dSAX microscope system. (d)-(f) are the 1D profiles of the low-pass filter corresponding to (a)-(c), obtained at the dotted line in (a).

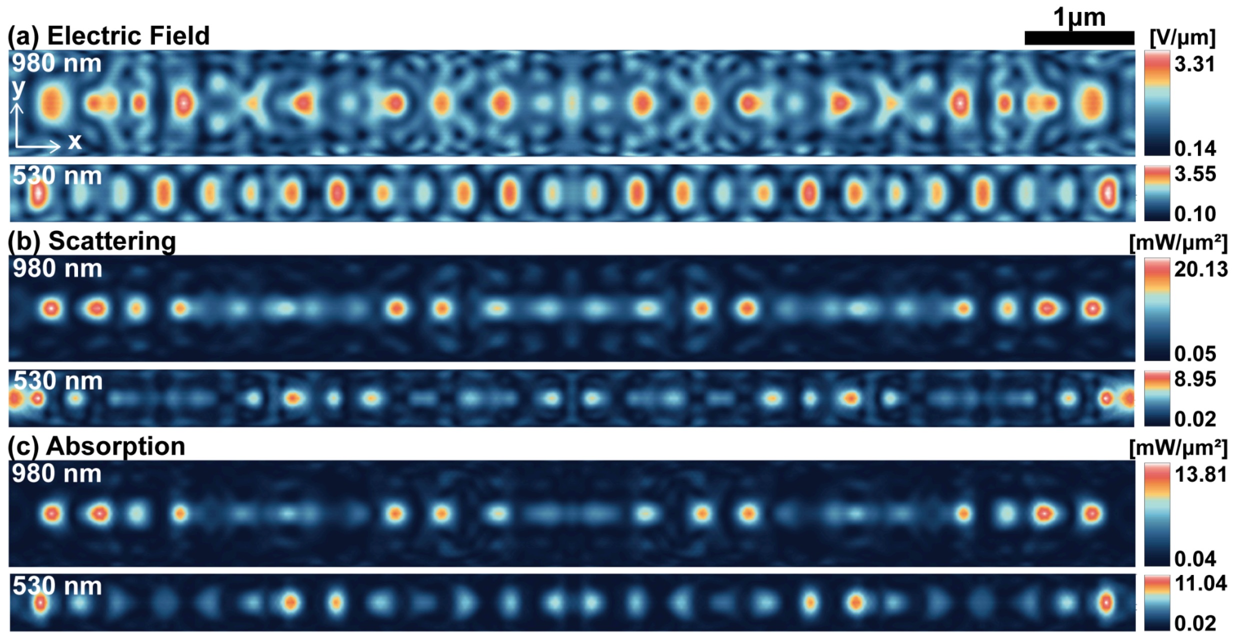

Figure S7. FEM simulation on the optical response of 980 nm and 530 nm diameter silicon nitride nanowire under single wavelength wide-field illumination. (a-c) Two-dimensional calculated mapping of electric field intensity (a), scattering (b), and absorption intensity (c). The length of the nanowire is 10.7 µm. The excitation wavelength is 561 nm, and the excitation intensity is 1 mW/µm^2.

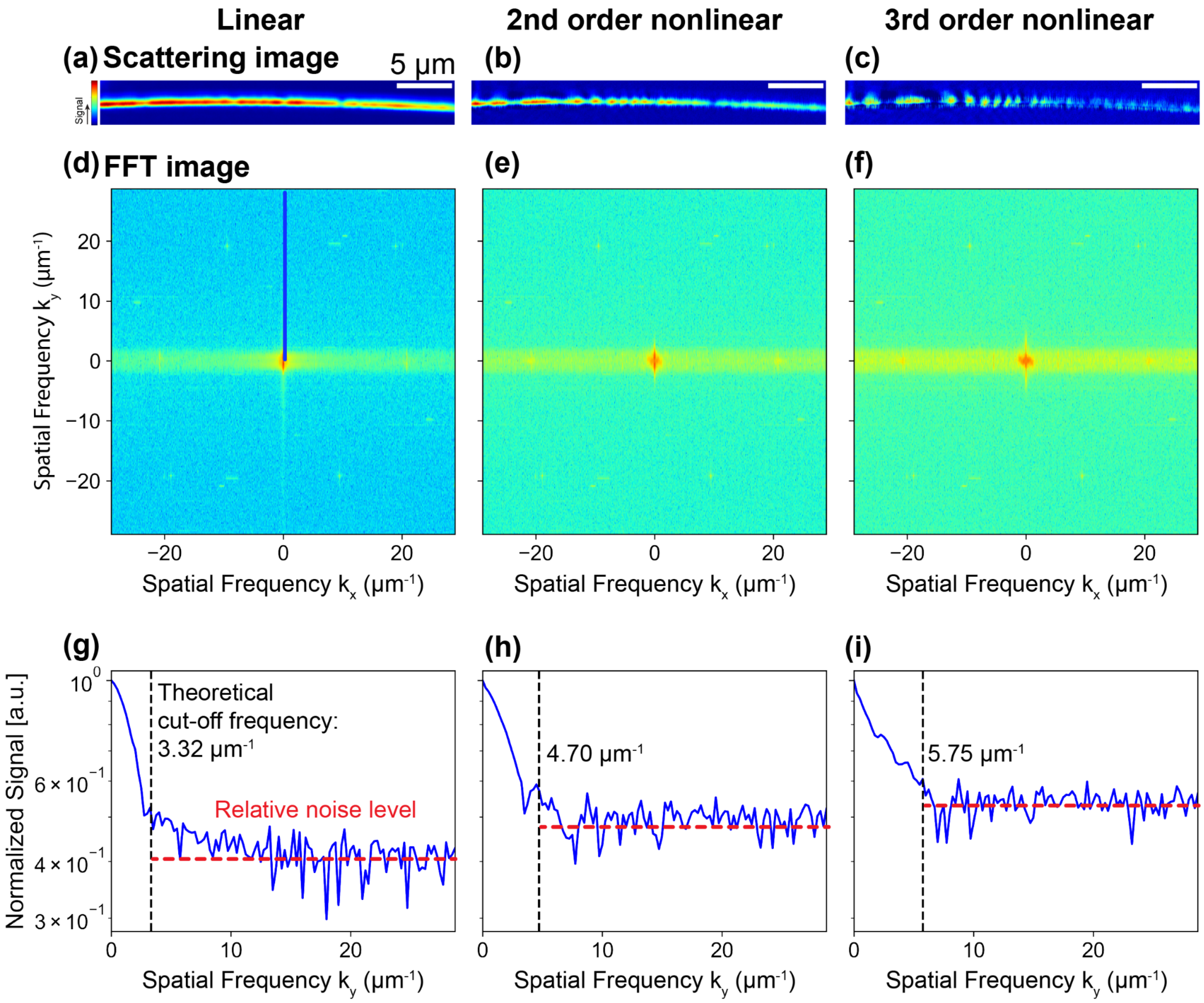

Figure S8. Image analysis in spatial frequency domain. (a-c) dSAX scattering image of Si_3N_4 nanowire (diameter = 530 nm) reconstructed by linear (a), 2nd order (b) and 3rd order nonlinear scattering signals (c). (d-f) Fourier transform images of (a-c). (g-i) Normalized signal profiles of (d-f) obtained at the location of the solid line in (d). The black dotted line indicates the theoretical cut-off spatial frequencies, respectively corresponding to 3.32, 4.70, and 5.75 μm^{-1}, for linear, 2nd order and 3rd order nonlinear imaging in our dSAX microscopy setup. The red dotted lines are the relative noise levels estimated by the profiles, which corresponds to 0.40 (g), 0.50 (h) and 0.55 (i).

Supporting Video 1. FEM simulation on the electric field of Si_3N_4 nanowire under laser scanning through the wire axis (x-axis). (a-b) Calculated electric field strength in the xy plane of the wire center (a), and xz plane of the wire center (b). The diameter and the length of the nanowire are 276 nm and 8.7 μm, respectively. The excitation wavelength is 561 nm, and the excitation intensity is 1 mW/μm^2. The incident electric field is a linearly y-polarized paraaxial-approximation NA=0.95 Gaussian beam propagating in the direction of +z-axis. The black hollow circle and arrow indicate the excitation position and direction. The distance between the left end of the wire and the incident position is written on the top of (a). The wire position is marked by the white dashed rectangle. (a) and (b) use the same scale bar shown in (a).

REFERENCES

(1) Bohren, Craig F., and Donald R. Huffman. Absorption and scattering of light by small particles. *John Wiley & Sons*, 2008.

(2) Aaron C. Hryciw, Rohan D. Kekatpure, Selçuk Yerci, Luca Dal Negro, Mark L. Brongersma, Thermo-optic tuning of erbium-doped amorphous silicon nitride microdisk resonators. *Appl. Phys. Lett.* **2011**, *98*, 041102.

(3) Leonid Yu. Beliaev, Evgeniy Shkondin, Andrei V. Lavrinenko, Osamu Takayama, Optical, structural and composition properties of silicon nitride films deposited by reactive radio-frequency sputtering. *Thin Solid Films*, **2022**, 763, 139568.

(4) Andrew T. Land, Mitul Dey Chowdhury, Aman R. Agrawal, Dalziel J. Wilson, Sub-ppm nanomechanical absorption spectroscopy of silicon nitride. *Nano Letters*. **2024**, *24*, 7578-7583.
